# Phenotype Recognition with Combined Features and Random Subspace Classifier Ensemble

**DOI:** 10.1186/1471-2105-12-128

**Published:** 2011-04-30

**Authors:** Bailing Zhang, Tuan D Pham

**Affiliations:** 1 Department of Computer Science and Software Engineering, Xi'an Jiaotong-Liverpool University, Suzhou, 215123, P.R.China; 2 School of Engineering and Information Technology, The University of New South Wales, Canberra, ACT 2600, Australia

## Abstract

**Background:**

Automated, image based high-content screening is a fundamental tool for discovery in biological science. Modern robotic fluorescence microscopes are able to capture thousands of images from massively parallel experiments such as RNA interference (RNAi) or small-molecule screens. As such, efficient computational methods are required for automatic cellular phenotype identification capable of dealing with large image data sets. In this paper we investigated an efficient method for the extraction of quantitative features from images by combining second order statistics, or Haralick features, with curvelet transform. A random subspace based classifier ensemble with multiple layer perceptron (MLP) as the base classifier was then exploited for classification. Haralick features estimate image properties related to second-order statistics based on the grey level co-occurrence matrix (GLCM), which has been extensively used for various image processing applications. The curvelet transform has a more sparse representation of the image than wavelet, thus offering a description with higher time frequency resolution and high degree of directionality and anisotropy, which is particularly appropriate for many images rich with edges and curves. A combined feature description from Haralick feature and curvelet transform can further increase the accuracy of classification by taking their complementary information. We then investigate the applicability of the random subspace (RS) ensemble method for phenotype classification based on microscopy images. A base classifier is trained with a RS sampled subset of the original feature set and the ensemble assigns a class label by majority voting.

**Results:**

Experimental results on the phenotype recognition from three benchmarking image sets including HeLa, CHO and RNAi show the effectiveness of the proposed approach. The combined feature is better than any individual one in the classification accuracy. The ensemble model produces better classification performance compared to the component neural networks trained. For the three images sets HeLa, CHO and RNAi, the Random Subspace Ensembles offers the classification rates 91.20%, 98.86% and 91.03% respectively, which compares sharply with the published result 84%, 93% and 82% from a multi-purpose image classifier WND-CHARM which applied wavelet transforms and other feature extraction methods. We investigated the problem of estimation of ensemble parameters and found that satisfactory performance improvement could be brought by a relative medium dimensionality of feature subsets and small ensemble size.

**Conclusions:**

The characteristics of curvelet transform of being multiscale and multidirectional suit the description of microscopy images very well. It is empirically demonstrated that the curvelet-based feature is clearly preferred to wavelet-based feature for bioimage descriptions. The random subspace ensemble of MLPs is much better than a number of commonly applied multi-class classifiers in the investigated application of phenotype recognition.

## Background

Complex cellular structures such as molecular construction of a cell can be studied by fluorescence microscopy images of cells with appropriate stains. Robotic systems nowadays can automatically acquire thousands of images from cell assays, which are often referred as being "high-content" for the large amount of information. These images reflect the biological properties of the cell with many features, including size, shape, amount of fluorescent label, DNA content, cell cycle, and cell morphology. With interdisciplinary efforts from computer science and biology, scientists are now able to carry out large-scale screening of cellular phenotypes, at whole-cell or sub-cellular levels, which are important in many applications, e.g., delineating cellular pathways, drug target validation and even cancer diagnosis [1,2].

From the high-throughput screening, biologists can also greatly benefit in further understanding the complex cellular processes and genetic functions [3,4]. For example, a gene's normal operations in the cell can be assessed by observing the downstream effect of perturbing gene expression [5]. By introduction of double-stranded RNA (dsRNA) into a diverse range of organisms and cell types, the complementary mRNA can be degraded, a phenomenon known as RNA interference (RNAi) [6,7]. The discovery of RNAi and the availability of whole genome sequences allow the systematic knockdown of every gene or specific gene sets in a genome [8-10]. Image-based screening of the entire genome for specific cellular functions thus becomes feasible by the development of Drosophila RNAi technology to systematically disrupt gene expression [11,12]. Genome-wide screens, however, produce huge volumes of image data which is beyond human's capability of manual analysis, and automating the analysis of the large number of images generated in such screening is the bottleneck in realizing the full potential of cellular and molecular imaging studies.

To advance the development of high content screening (HCS) for genome analysis, computer vision and pattern analysis techniques have to be resorted to characterize morphological phenotypes quantitatively and to identify genes and their dynamic relationships on a genome-wide scale [4,13]. Such a bio-image informatics framework would consist of several components: cellular segmentation, cellular morphology and image feature extraction, cellular phenotype classification, and clustering analysis [11,14]. In this article, our effort is made toward further investigating the challenging multi-class phenotype classification problem from microscopy images by using benchmarking fluorescence microscopy images [15-17].

With appropriate cellular segmentation results, phenotype recognition can be studied in a multi-class classification framework, which involves two interweaved components: feature representation and classification. Efficient and discriminative image representation is a fundamental issue in any bioimage recognition tasks. Most of the proposed approaches for image-based high-content screening employed feature set which consist of different combinations of morphological, edge, texture, geometric, moment and wavelet features [15,18-22]. In recent years, computer science has seen much progresses in the development of various efficient image feature description methods, many of which have become "off-the-shelf" standard techniques applicable to bioimages analysis. In this paper, we will show that the curvelet transform [23-27] outperform many other known feature descriptions for the cellular images. Based on the latest research achievements on multiresolution analysis for image, Curvelet Transform can accurately capture edge information by taking the form of basis elements which exhibit very high directional sensitivity and are highly anisotropic. It has been shown that curvelet is well suited for representing images which are rich of edge information and the efficiency has been demonstrated in many tasks [28-31].

To our knowledge, there is no previous work discussing the use of Curvelet transform in fluorescence microscope images except [32] which applied curvelet for denoising. The research presented in this paper is to investigate the application of curvelet on microscope images based phenotype recognition, and compare it with the application of wavelets. The simple statistics of mean and standard deviation from multiscale curvelet transform coefficients are extracted and evaluated as the basic curvelet features. On the other hand, due to the proven effectiveness of the traditional Haralick features [33] for extracting texture information, and in microscope images of biological cells in particular [18,19], we proposed to form a combined image description from the Curvelet Transform and Haralick features. Haralick feature is based on gray-level spatial dependencies using a Gray Level Co-occurrence Matrix (GLCM) that measures the frequency that a particular gray level is found adjacent to another gray level. By complementarily combining the advantages of each feature description method, the classification performance can be considerably enhanced.

Machine learning methods such as artificial neural networks and Support Vector Machine (SVM) have been utilized for the classification of subcellular protein location patterns with fluorescence microscope images [18-21,34]. Multi-class phenotype images, however, are often featured with large intra-class variations and inter-class similarities, which poses serious problems for simultaneous multi-class separation using the standard classifiers. And other rate-limiting factors challenging classifier design is that the dimension of the feature space is often larger than the number of training examples per class. In recent years, many sophisticated learning paradigms have been proposed to design accurate and robust classification systems. Among them, ensemble learning has attracted much attention due to the promising results for many applications. An ensemble of classifiers integrates multiple base classifiers such as multiple layer perceptrons (MLP) using a same learning algorithm [35,36]. Among the representatives of ensemble learning, Random Subspace method (RSM) [37] is an efficient way to create ensemble of classifiers. RSM divides the entire space of features into subspaces and each subspace is formed by randomly picking features from the entire space, allowing for features to be repeated across subspaces. In this paper, we propose to construct and evaluate a Random Subspace classifier ensemble with multiple layer perceptron as the base classifier, using the combined features from Curvelet Transform and Haralick features.

## Results

Three image sets HeLa, CHO and RNAi from the IICBU benchmarking data [15,16] were evaluated. All of the images are processed as wholes without any detection or segmentation. In our experiments, a simple holdout methodology was applied. For each of the three image sets, we randomly split it into training and test sets, each time with 20% of each class's images reserved for testing while the rest for training. The classification results are the average from 100 runs, such that each run used a random split of the data to training and test sets.

**Figure 1:**
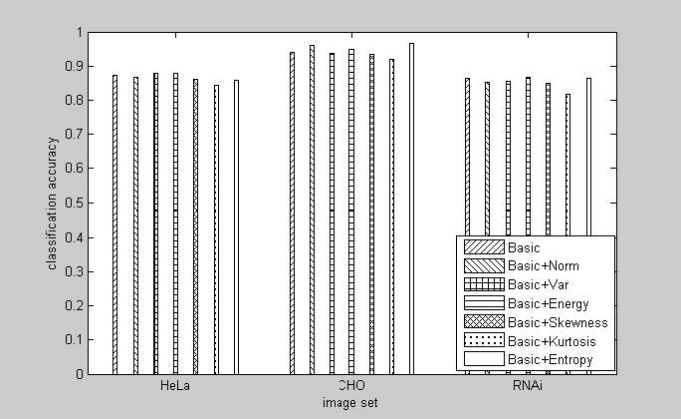
**Comparison of the performance for different curvelet-based feature vectors for phenotype classifications from HeLa, CHO and RNAi images**.

Our first set of experiments aims at selecting appropriate features from the curvelet transform. The simple statistics of mean and standard deviation from each band of curvelet transform have been used as efficient signatures in several applications [29-31]. Rather than theoretical analysis of the distribution properties of Curvelet Transform coefficients for the cell images, we conducted empirical analysis for the usefulness to include other statistics into the feature vectors, for example, energy, entropy, skewness and kurtosis. To determine the discriminating power from different combinations of these statistics as the image features, we compared the classification performance from the following candidate curvelet feature vectors. In addition to the basic signature of mean and standard deviation (basic), other sets of statistics were calculated and added to the basic signature in turn, including norm, energy, variance, skewness, kurtosis, and entropy. The results were reported in Figure 1. While the addition of variance or energy slightly increase the classification accuracy for the HeLa images, the inclusion of entropy clearly outperform other kind of combinations for both of the CHO and RNAi images.

**Figure 2:**
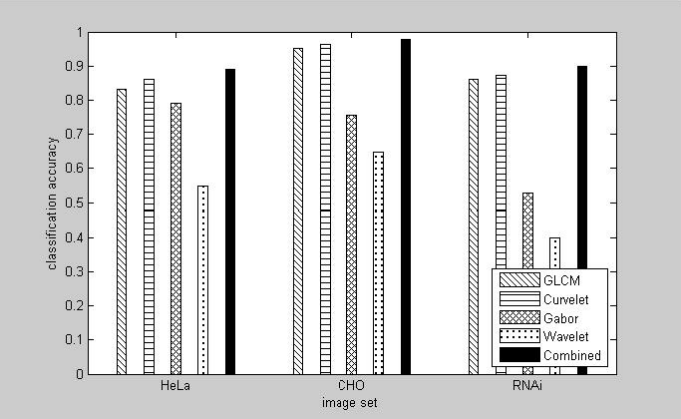
**Comparison of the Daubechies wavelet-based, Gabor-wavelet based, GLCM-based and curvelet-based feature descriptors for phenotype classifications from HeLa, CHO and RNAi images**.

The discriminating strengths of the feature vectors from GLCM [33], Daubechies wavelet [38], Gabor wavelet [39,40] and Curvelet transform [23] were compared using three-layer perceptron (MLP) neural network, which has the number of inputs same as the number of features, one hidden layer with 20 units and linear units representing the class labels (10 for HeLa and RNAi, 5 for CHO). The networks are trained using the Conjugate Gradient learning algorithm for 500 epochs. Figure 2 illustrates the comparison of accuracies for each of the three image sets from GLCM, Daubechies wavelet, Gabor wavelet, and curvelet feature descriptors. For all of the three image sets, the curvelet descriptors clearly outperforms all other descriptors. Specifically, for HeLa, CHO and RNAi images, the classification accuracies from Curvelet features are 86.1%, 96.4% and 87.3% respectively, which compares favorably over the results from GLCM features 83.3%, 95.2%, and 86%. Among the four different features compared, the curvelet features also consistently demonstrates the superiority over Gabor wavelet and Daubechies wavelet, which were in fact expected since the curvelet transform is able to capture the multi-dimensional features in wedges as opposed to points in wavelet transforms. The multidirectional features in curvelets prove to be very effective in the classification of microscopy cell images which often demonstrate piece-wise smooth with rich edge information. The best classification accuracies for all of the three image sets were from the aggregated features by simply concatenating curvelet and GLCM, namely, HeLa 89.2%, CHO 98.2% and RNAi 89.9% respectively. The benefit of applying them in an integrative way is due to the fact that GLCM and curvelet transform features emphasize texture and edge or piece-wise smooth characteristics of image differently. As expected, the combination of both features from GLCM and curvelet transform produces a higher accuracy than the methods being used singly, as shown in Figure 2.

We proceeded to evaluate several different but commonly used supervised learning methods to the multi-class phenotype classification problem, including *k*-nearest neighbors (*k*NN), multi-layer perceptron neural networks, SVM and random forest, using the above three image sets. The feature vector for each image is calculated from the sub-bands of curvelet transform (Basic + Variance for HeLa, Basic + Entropy for CHO and RNAi). We simply chose *k *= 1 for *k*NN in the comparisons. MLP is the same as used in the above experiment. Designing SVM classifiers [41] includes selecting the proper kernel function and choosing the appropriate kernel parameters and *C *value. The popular library for SVM, LIBSVM http://www.csie.ntu.edu.tw/~cjlin/libsvm, was used in the experiment. We used the radial basis function kernel for the SVM classifier. The parameter γ that defines the spread of the radial function was set to be 5.0 and parameter *C *that defines the trade-off between the classifier accuracy and the margin (the generation) to be 3.0. A random forest (RF) classifier [42] consists of many decision trees and outputs the class that is the mode of the classes output by individual trees. In the comparison experiments, the number of trees for random forest classifier was chosen as 300 and the number of variables to be randomly selected from the available set of variables was selected as 20.

**Figure 3:**
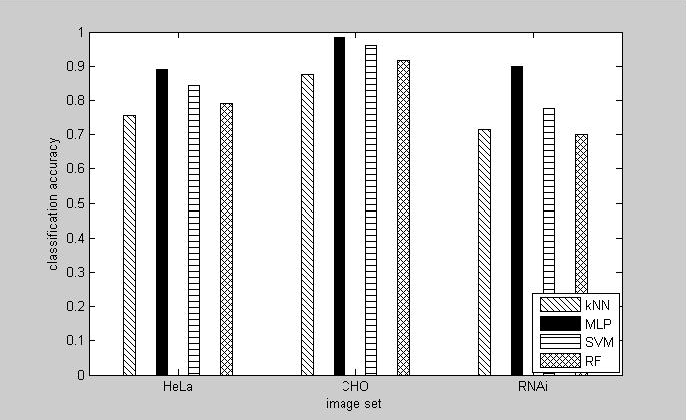
**Comparison of the performance for four different classifiers using the composite features from curvelet transform and GLCM**.

The comparison results from applying the above four classifiers were provided in Figure 3, which confirmed that for each image dataset, the best result was obtained by using MLP. For RNAi, the result from MLP is 89.9%, which is better than the published result 82% [15]. The accuracies from other three classifiers are 71.6% (*k*NN), 70.1% (random forest), and 77.5% (SVM). For 2D-Hela and CHO, the correct classification rates from MLPs are 89.2% and 98.4%, respectively, which are also very competitive. The results for these two datasets obtained by Shamir et al. are 84% for 2D-Hela and 93% for CHO [15]. The results obtained by MLP contrast to the generally accepted perception that SVM classifier is better than neural network in classification. The most reasonable explanation for the better performance of MLP from our experiment is that MLP as a memory-based classifier is more resistant to insufficient data amount comparing the margin or distance-based SVM.

Our next experimental study aims to demonstrate the superiority of Random Subspace MLP classifier ensemble over the single MLP used in the previous experiments. The holdout experiment setting is similar. To ensure the diversity among the MLPs in an ensemble, the numbers of hidden units in the component networks are varied by randomly choosing them from a range of 30 ~50. Using an ensemble of size 20 and 80% of original dimensionality for feature subsets, the classification results obtained for HeLa, CHO and RNAi were summarized in Table 1, from which one can see that for all the three image sets, the random subspace MLP ensemble does bring the improvement on the classification accuracy.

**Table 1:** Performance Improvement from Random Subspace Ensemble (RSE)

Classifier	RNAi	2D-Hela	CHO
MLP	89.90%	89.20%	98.02%

MLP-RSE (ensemble size = 20)	90.10%	90.65%	98.40%

In the Random Subspace method, there are two important parameters that have to be considered. The first is the ensemble size *L*, which is the number of base classifiers in the ensemble. The second is the dimensionality of feature subsets *M*. Recently, the selection of these two parameters has been addressed in the classification of brain images of fMRI [36] and text categorization [43], which shows that relatively medium *M *and small *L *yield an ensemble that could improve the performance. Our next experiment assessed the effect of ensemble size for the phenotype image classifications. We first varied the sizes of the ensembles from 5 components MLPs to 50, with fixed feature subspace dimensionality 350, which account for 80% of combined features' dimension. The same experiment procedure described above was repeated. The results of the averaged classification accuracies in Table 2 shows that there are no real benefits of forming very large ensembles. In the case of RNAi, the maximum accuracy 91.03% is reached with size 5, beyond which there is no improvement in performance. In the cases of HeLa and CHO, size 15 seems to be sufficient for the much increased classification accuracies comparing to the results in [15]. Beyond size 15, larger ensemble size may bring quite marginal improvement for the CHO images at the cost of heavy computational burden in the training phase.

**Table 2:** Comparison of Classification Performance of Varying Ensemble Sizes

Size	5	10	15	20	25	30	35	40	45	50
RNAi	91.03%	90.37%	90.05%	90.10%	89.62%	90.05%	89.38%	90.34%	89.27%	89.50%

HeLa	89.99%	90.52%	90.94%	90.65%	90.59%	90.45%	90.90%	91.05%	90.38	90.07%

CHO	98.34%	98.34%	98.52%	98.40%	98.34%	98.74%	98.60%	98.64%	98.46%	98.46%

To answer the question how the dimensionality of feature subsets *M *influence the classification performance of RSM, we compared the RSM classification performances for the three image sets by varying subspace dimensionalities. For original 438 dimensional combined features, selection of 55% features means 241 dimensionality and so on. With 40 component MLPs in the ensemble, the comparison results are given in Table 3, which indicates that satisfactory results are obtained with medium *M*s. For the RNAi data, maximum 90.34% classification rate is reached with selection of 80% of original features (*M *= 350). For the HeLa and CHO image sets, the largest accuracies 91.20% and 98.86% are from selecting 75% and 85% of original features (*M *= 328 and *M *= 372), respectively.

**Table 3:** Comparison of Classification Performance from Random Subspace Ensemble by Varying the Subspace Dimensionalities

Dimensionalities(%)	55	60	65	70	75	80	85	90	95	100
RNAi	89.34%	90.10%	90.20%	90.60%	89.90%	90.34%	90.20%	89.50%	89.34%	89.74%

HeLa	90.49%	90.23%	90.52%	90.57%	91.15%	90.85%	91.00%	90.51%	90.66%	90.70%

CHO	98.58%	98.68%	98.50%	98.46%	98.64%	98.64%	98.86%	98.30%	98.50%	98.80%

The confusion matrices that summarize the details of the above random subspace ensemble for the three image sets are given in the following Tables 4, 5 and 6. In the confusion matrix representation, the rows and columns indicate true and predicted class, respectively. The diagonal entries represent correct classification while the off-diagonal entries represent incorrect ones. Confusion matrix is often used in the study of multi-class classification problems and to measure the similarity between classes of phenotypes [44]. For the HeLa images, the RS ensemble can well distinguish two Golgi proteins, GPP130 and giantin, which have been shown to be very difficult to discriminate by visual inspection [45]. For the RNAi image sets, it is apparent that among the 10 classes, *CG10873 *type is the easiest to be correctly classified while the *CG9484 *is the most difficult one. On the other hand, the resulting confused genes do not directly share the related biological mechanisms. Each of these genes is associated with a different mechanism, but some pathways can be more similar to each others than others. For example, the gene CG8114 (Pebble), which leads to binucleate phenotype, is sometimes confused with gene CG3938 (CyclinE), which is associated with G1 arrest. These two genes are more related to each other than other pairs and the confusion is often expected. However, the results from Random Subspace ensemble indicate that these two genes can be well distinguished without any confusion.

**Table 4:** Averaged confusion matrix for RNAi (**%**)

	CG10873	CG1258	CG3733	CG7922	CG8222	CG12284	CG17161	CG3938	CG8114	CG9484
CG10873	100	0	0	0	0	0	0	0	0	0
CG1258	0	90.39	0	0	0	0	9.61	0	0	0
CG3733	0	0	98.76	0	0	0	0.99	0.25	0	0
CG7922	0	0	0	98.36	0	0	0	1.64	0	0
CG8222	0	5.04	0	0	89.12	0	1.59	0.27	0	3.98
CG12284	0	2.42	0	0	0.24	86.96	2.42	1.21	3.86	2.90
CG17161	0	9.28	1.59	0	0.27	0	88.86	0	0	0
CG3938	1.90	0	0	6.50	1.90	0.27	0	89.43	0	0
CG8114	0	0	0	0	0	1.11	0	0	98.89	0
CG9484	3.40	0	0.24	3.88	12.14	8.74	0	0	1.7	69.90

**Table 5:** Averaged confusion matrix for HeLa (**%**)

	Actin	TfR	ER	Giantin	GPP130	LAMP2	Tubulin	**Mitoch**.	Nucleolin	DNA
Actin	100	0	0	0	0	0	0	0	0	0
TfR	0	80.36	1.25	0	0.23	10.78	3.75	3.63	0	0
ER	0	0	93.81	0	0	0	0.80	3.78	0	1.61
Giantin	0	0	0	95.56	4.21	0	0	0.23	0	0
GPP130	0	0.24	0.84	10.79	87.17	0.12	0	0	0.84	0
LAMP2	0	7.54	0	0.75	1.88	86.43	0	1.63	1.76	0
Tubulin	0.34	5.82	8.73	0	1.90	0	80.40	2.69	0	0.11
Mitoch.	0	4.04	3.35	0	1.81	2.09	0.42	88.15	0	0.14
Nucleolin	0	0	0	0.48	0.12	0	0	0.12	99.28	0
DNA	0	0	1.61	0	0	0	0	0	0	98.39

**Table 6:** Averaged confusion matrix for CHO (**%**)

	anti-giantin	DNA	anti-lamp2	anti-nop4	anti-tubulin
anti-giantin	99.73	0	0	0	0.27
DNA	0	100	0	0	0
anti-lamp2	0	0	100	0	0
anti-nop4	0	0	0	98.34	1.66
anti-tubulin	1.70	0	0	4.67	93.63

## Discussion

Accurately and robustly classification of cellular phenotypes is still a challenging task in image based high-content screening. To find the best description for the microscopy images, many features extraction methods have been attempted in previous studies, but all encounter with various aspects of difficulty in dealing with various irregularities in the cellular morphology.

Our motivation for overcoming this challenge is two-fold. Firstly, we have attempted to demonstrate that the classification can be improved by using highly discriminative image features. This can be achieved by the utilization of the curvelet transform to extract such a feature. Being similar to various wavelet transforms, the curvelet transform can capture the structural information of the images in this study at multiple scales, locations, and orientations. The major advantage is that the curvelet transform can identify the structural detail along the radial 'wedges' in the frequency domain, which is inherent in images of rich edge information. Secondly, the notion of combination of multiple complementary features can further improve the classification performance. The combination of features for pattern classification has recently been embraced by the research community of image processing and pattern recognition [46]. However, most proposed methods for feature combination are based on optimization techniques or machine learning algorithms, which are often very complicated for practical implementation. Our proposed method offers a much simpler solution by transforming multiple features into a single representation based on which the random subspace classifier ensemble can efficiently integrate different aspects of information from various random subspaces. By using this strategy, the high dimensionality problem arising from using the conventional combination of multiple features is therefore implicitly avoided.

In general, the method we have proposed in this paper can be applied to classifying and understanding complex patterns of many biological systems. The gaining of such knowledge can quickly provide life-science researchers with new insights into the changing biological behaviors in various treatment conditions, and to facilitate rapid screening and testing of new therapeutic interventions for major public health problems.

## Conclusion

This paper further investigated the challenging multi-class phenotype classification problem from microscopy images. Two contributions are presented. Firstly, we proposed to combine two different feaures from Gray Level Co-occurrence Matrix (GLCM) and curvelet transform to efficiently describe microscopy images. Secondly, we examined random subspace classifier ensemble with multi-layer perceptron as the base classifier, which seem to be well-suited to the characteristics of microscopy images, as the high dimensionality of the data could be implicitly solved by randomly selecting subsets of features. Experiments on three benchmarking microscopy image datasets showed that the random subspace MLP ensemble method achieved significantly higher classification accuracies (91% for RNAi, 91.2% for HeLa and 98.9% for CHO), compared to the published results (82% for RNAi, 84% for HeLa and 93% for CHO), which used wavelet as part of the features and a general-purpose classification scheme WND-CHARM. This supports the claim that Random Subspace ensemble can be used as a simple yet efficient classifier design methodolody and curvelet features effectively measure the informativeness in the microscopy images.

## Methods

### The Benchmarking Fluorescence Microscopy Image Datasets

Three benchmark fluorescence microscopy image datasets in [15,16] were used in our study, which are 2D-Hela, CHO and RNAi. The 2D HeLa dataset is a collections of HeLa cell immunofluorescence images containing 10 distinct subcellular location patterns. The 10 organelles from the images are DNA (Nuclei), ER (Endoplasmic reticulum), Giantin, (cis/medial Golgi), GPP130 (cis Golgi), Lamp2 (Lysosomes), Mitochondria, Nucleolin (Nucleoli), Actin, TfR (Endosomes), Tubulin. CHO is a dataset of fluorescence microscope images of CHO (Chinese Hamster Ovary) cells. The images were taken using 5 different labels. The labels are: anti-giantin, Hoechst 33258 (DNA), anti-lamp2, anti-nop4, and anti-tubulin.

The RNAi dataset is a set of fluorescence microscopy images of fly cells (*D. melanogaster*) subjected to a set of gene-knockdowns using RNAi. The cells are stained with DAPI to visualize their nuclei. Each class contains 20 1024 × 1024 images of the phenotypes resulting from knockdown of a particular gene. Ten genes were selected, and their gene IDs are used as class names. The genes are CG1258, CG3733, CG3938, CG7922, CG8114, CG8222, CG 9484, CG10873, CG12284, CG17161 [15,16]. According to [15,16], the images were acquired automatically using a Delta-Vision light microscope with a 60× objective. Each image is produced by deconvolution, followed by maximum intensity projection (MIP) of a stack of 11 images at different focal planes.

**Figure 4:**
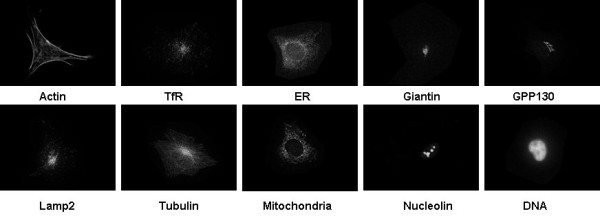
**Sample 2D HeLa images**.

**Figure 5:**
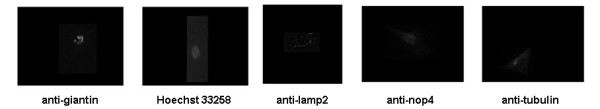
**Sample CHO images**.

**Figure 6:**
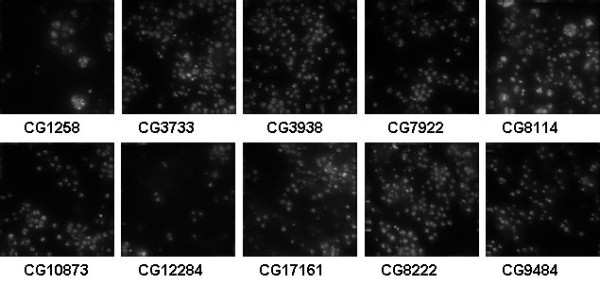
**RNAi image set of fluorescence microscopy images of fly cells (D. melanogaster)**.

Samples of the above three image sets are illustrated in Figures 4, 5 and 6, respectively.

### Image Feature Extraction

Once the cell images are segmented, various feature extraction methods can be applied. In our study, we calculated texture features using the co-occurrence matrices (Haralick features) and applied three multi-resolution transforms, including Daubechies wavelet, Gabor wavelet and curvelet.

#### Gray Level Co-occurrence Matrices

Gray level co-occurrence matrix (GLCM) proposed by Haralick [33] is a common texture analysis method which estimates image properties related to second-order statistics. GLCM matrix is defined over an image to be the distribution of co-occurring values at a given offset. Mathematically, a co-occurrence matrix *C *is defined over an *n *× *m *image *I*, parameterized by an offset (Δ*x*, Δ*y*) as(1)

Note that the (Δ*x*, Δ*y*) parameterization makes the co-occurrence matrix sensitive to rotation. An offset vector can be chosen such that a rotation of the image not equal to 180 degrees will result in a different co-occurrence distribution for the same image.

In order to estimate the similarity between different GLCM matrices, many statistical features can be extracted from them. The most relevant features that are widely used in literature include: (1). Energy, which is a measure of textural uniformity of an image and reaches its highest value when gray level distribution has either a constant or a periodic form; (2). Entropy, which measures the disorder of an image and achieves its largest value when all elements in *C *matrix are equal; (3). Contrast, which is a difference moment of the *C *and measures the amount of local variations in an image. In addition to these standard features, we also calculated the following features [33,47,48] derivable from a normalized co-occurrence matrix:

• Correlation

• Cluster Prominence

• Cluster Shade

• Homogeneity

• Sum of sqaures

• Sum variance

• Sum entropy

• Difference variance

• Inverse difference (INV)

• Inverse difference normalized (INN)

The details of these textbook materials are not included here as they can be found in many resources, for example [47,48].

For GLCM feature case, 16 gray co-occurrence matrices were created for each image with an offset that specifies four orientations 0, *π*/4, *π*/2 and 3*π*/4 and 4 distances (1,2,3 and 4 pixels) for each direction. Then for each normalized co-occurrence matrix *P *(*i*, *j*), 12 different type of statistic measurements were estimated, including correlation, variance, contrast, energy, difference variance, entropy, and homogeneity. Thus the dimension of GLCM feature is 16 × 12 = 192.

#### Discrete Wavelet Transform

Wavelet transform [38] has some nice features of space-frequency localization and multi-resolutions. Let *L*^2^(*R*) denote the vector space of a measurable, square integrable, one-dimensional function. The continuous wavelet transform of a 1D signal *f*(*t*) ∈ *L*^2^(*R*) is defined as(2)

where the wavelet basis function *ϕ*_*a*, *b*_(*t*) ∈ *L*^2^(*R*) can be expressed as(3)

These basis functions are called wavelets and have at least one vanishing moment. The arguments *a *and *b *denote the scale and location parameters, respectively. The oscillation in the basis functions increases with a decrease in *a*. Eq. (2) can be discretized by restraining *a *and *b *to a discrete lattice (*a *= 2*^n^*, *b *∈ *Z*). Typically, there are some more constraints on *ϕ *when a non-redundant complete transform is implemented and a multiresolution representation is pursued.

The wavelet basis functions in Eq.(3) are dilated and translated versions of the mother wavelet *ϕ *(*t*). Therefore, the wavelet coefficients of any scale (or resolution) could be computed from the wavelet coefficients of the next higher resolutions. This enables the implementation of wavelet transform using a tree structure known as a pyramid algorithm [38]. Here, the wavelet transform of a 1D signal is calculated by splitting it into two parts, with a low-pass filter (LPF) and high pass filter (HPF), respectively. The low frequency part is split again into two parts of high and low frequencies. And the original signal can be reconstructed from the DWT coefficients. The DWT for two dimensional images *x*[*m*, *n*] can be similarly defined by implementing the one dimensional DWT for each dimension *m *and *n *separately: *DWT_n_*[*DWT_m_*[*x*[*m*, *n*]]]. 2D Wavelet transform (WT) decomposes an image into "subbands" that are localised in frequency and orientation. A wavelet transform is created by passing the image through a series of filter bank stages.

There are several ways of generating a 2D wavelet transform. The construction of the digital filters differs mainly in their scaling and wavelet coefficients. Scaling and wavelet function coefficients are characteristic of their particular families. In the following, we'll use Daubechies D4 for image decomposition [38]. The Daubechies (D4) transform has four wavelet and scaling coefficients. In our application, two levels of resolution were extracted for each wavelet. At each resolution level, the wavelet has three detail coefficient matrices representing the vertical, horizontal and diagonal structures of the image. From each of the detail coefficient matrices, the first-order and second-order statistics mean and standard deviation were calculated as image features.

#### Gabor Wavelet

Gabor wavelets [39,40] are often used to construct spectral filters for segmentation or detection of certain image texture and periodicity characteristics. In [15,16], Gabor wavelet has been used as one of the main features for image representation of cell microscope images. The convolution kernel of Gabor filter is a product of a Gaussian and a cosine function, which can be characterized by a preferred orientation and a preferred spatial frequency:(4)

where

The standard deviation *σ *determines the effective size of the Gaussian signal. The eccentricity of the convolution kernel *g *is determined by the parameter *λ*, called the spatial aspect ratio. *λ *determines the frequency (wavelength) of the cosine. *θ *determines the direction of the cosine function and finally, *φ is the phase offset.

Typically, an image is filtered with a set of Gabor filters of different preferred orientations and spatial frequencies that cover appropriately the spatial frequency domain, and the features obtained form a feature vector that is further used for classification. Given an image *I*(*x*, *y*), its Gabor wavelet transform is defined as(5)

where * indicates the complex conjugate. We assume the local texture regions are spatially homogeneous. The mean *μ_mn _*and standard deviation *σ_mn _*of the magnitude of transform coefficients are used to represent the regions for classification:(6)(7)

The Gabor feature vector contains pairs for all the scales and orientations of the wavelets. From a number of experiments we found that a filter bank with six orientations and four scales gave the best classification performance, which means 24 × 2 component features will be extracted for a given image patch. Therefore, the figuration is applied to 6 × 8 non-overlapping image subregions each with the size 60 × 64, yielding overall feature vector with length 4 × 5 × 48 = 960 for each image.

#### Curvelet Transform

**Figure 7:**
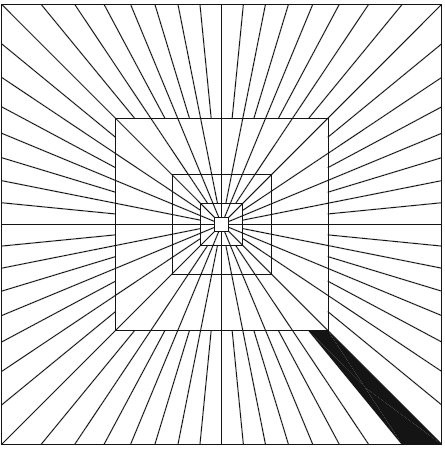
**Curvelet basic digital tiling**. The shaded region represents one such typical wedge.

Curvelet transform is a multiresolution geometric analysis proposed by Candes and Donoho [25] for the purpose of overcoming the drawbacks of conventional two-dimensional discrete wavelet transforms of lacking good representational capability for direction selectivity. The idea is to represent a curve as superposition of functions of various length and width obeying the curvelet scaling law *width *≈ *length*^2^[25]. Figure 7 presents the curvelet analysis method.

The needle-shaped elements of curvelets shown in Figure 7 possess very high directional sensitivity and anisotropy, which is quite different with the isotropic elements of wavelets [27]. Such elements are very efficient in representing line-like edge. Comparing the curvelet system with the conventional Fourier and wavelet analysis can further help our understanding. The short-time Fourier transform uses a shape-fixed rectangle in Fourier domain, and conventional wavelets use shape-changing (dilated) but area-fixed windows. By contrast, the curvelet transform uses angled polar wedges or angled trapezoid windows in frequency domain in order to resolve also directional features. The curvelet transform coefficients can be expressed by(8)

where *φ _j,l,k _*denotes curvelet function, and *j*, *l *and *k *denotes the variable of scale, orientation, and position respectively. In the frequency domain, the curvelet transform can be implemented with *φ *by means of the window function *U*. Defining a pair of windows *W *(*r*) (a radial window) and *V *(*t*) (an angular window) as below:(9)(10)

where variables *W *as a frequency domain variable, and *r *and *θ *for each *j *≥ *j*_0_, *U_j _*is defined in the Fourier domain by(11)

where [*j*/2] denotes the integer part of *j*/2.

From the curvelet coefficients, a common way to construct image descriptor is via some statistics calculated from each of these curvelet sub-bands. For example, the mean *μ *and standard deviation *δ *are the most convenient features that have been proven efficient in applications like image retrieval and face recognition [29-31]. If *n *curvelets are used for the transform, 2*n *dimensional feature vectors *G *= [*G_μ_*, *G_δ_*] are obtained, where *G_μ _*= [*μ*_1_, *μ*_2_, . . . , *μ_n_*], *G_δ_*= [*δ*_1_, *δ*_2_, . . . , *δ_n_*].

In this paper, we applied the second generation discrete curvelet transform [26], which is implemented in four steps: (1) using 2-D fast Fourier transform for the image; (2) forming the product of the scale and angle windows; (3) wrapping the aforesaid product around the origin; and (4) applying the 2-D inverse fast Fourier transform. The fast discrete curvelet transform via wedge wrapping was applied using the CurveLab Toolbox http://www.curvelet.org/ in the MATLAB development environment. Two parameters are involved in the implementation: number of resolutions and number of angles at the coarsest level. For the images of 2D HeLa, CHO and RNAi, five scales were chosen which include the coarsest wavelet level. At the 2nd coarsest level 16 angles were used. With 5 levels analysis, 82(= 1 + 16 + 32 + 32 + 1) subbands of curvelet coefficients were computed. Therefore, a 164 dimension feature vector was generated for each image in the three image sets.

#### Combined Features from GLCM and Curvelet Transform

Each feature extracted from above different methods characterizes certain aspect of image content. The joint exploitation of different image descriptions is often necessary to provide comprehensive discriptions in order for a classification system with higher accuracy. One of the difficulties of multiple feature aggregration lies in the high dimensionalities of the image features. However, with Random Subspace classifier ensemble which will be elaborated in the following, this problem is implicitly resolved due to its dimension reduction capability. Since the values of GLCM and curvelet features assume different ranges, first we normalize them in the range [-1, 1] before combining them in a single vector.

### Random Subspace Classifier Ensemble

A classifiers ensemble means a set of individually trained classifiers is integrated appropriately based on their component decisions [34,35]. Classifier ensembles generally offer improved performance due to the complementary information provided by the constituent classifiers. In this study, we consider artificial neural network as the base learners. The generalization performance of neural networks is not very stable in the sense that different settings such as different network architectures and initial conditions may all influence the learning outcome. The existing of such differences between base classifiers is a necessary condition to constitute classifier ensemble [34]. Multi-layer perceptron (MLP) has been successfully applied to the classification of subcellular protein location patterns [18,19] and the performance improvement can be expected from an MLP ensemble.

There are many ways to construct a classifier ensemble. In this paper we focus on MLP ensembles based on different feature subsets, following the principle of Random Subspace method (RSM) proposed in [37]. Being different with Bagging [49] and Boosting [50], not training samples but the feature variables from curvelet transform and GLCM are resampled, *i.e*., a large number of individual MLP models are trained on randomly chosen subsets of all available features (*i.e*. random sunspace). In another words, by dividing the entire space of features into subspaces, ensemble of MLP classifiers is created with one base classifier trained on each subspace from randomly selecting features from the entire space. The details of RS ensemble can be further elaborated as in the following.

Input: a *d*-dimensional labeled training data set

(1) Select a random projection from the *d*-dimensional feature space to a *k*-dimensional subspace;

(2) Project the data from the original *d*-dimensional feature space into the selected *k*-dimensional subspace;

(3) Train an MLP classifier on the acquired *k*-dimensional feature;

(4) Repeat steps 1-3 *m *times to obtain *m *different subspaces for the ensemble individuals;

(5) Aggregate the individual classifiers by majority voting.

## Authors' contributions

BZ made the contributions in investigating the proposed approach and conducting the experiments. TP played the role as a helper in the research with discussions on the details. Two authors have read and approved the final manuscript.

## Availability and Requirements

The reviewers can access the Matlab code, which has been included with the manuscript as additional file 1.

## Supplementary Material

Additional file 1 **Matlab code**. Matlab program used in the experiment, including feature extraction, different classifers comparison, and implementation of the random subspace ensemble, with the example of RNAi data. Click here for file
